# Editorial: The application of bioactive materials in bone repair

**DOI:** 10.3389/fbioe.2024.1539142

**Published:** 2025-01-06

**Authors:** Yori Endo, Elena Giunta, Jessica Mroueh, William McCarthy, Nina Graf

**Affiliations:** ^1^ Brigham and Women’s Hospital, Harvard Medical School, Boston, MA, United States; ^2^ Division of Hand, Plastic and Aesthetic Surgery, University Hospital, LMU Munich, Munich, Germany; ^3^ UCLA Extension, University of California, Los Angeles, Los Angeles, CA, United States

**Keywords:** bone repair, bioactive materials, scaffold, 3D printing, tissue engineering

The field of bioactive materials is revolutionizing bone repair by offering advanced solutions that not only mimic but also actively participate in biological processes to promote tissue regeneration. Unlike traditional bone repair materials, bioactive materials possess unique properties such as high biocompatibility, the ability to simulate the extracellular matrix, and the capacity for engineering modifications. These materials can form chemical bonds with bone tissue, enhancing bone healing at the molecular level and providing targeted, minimally invasive, and sustained therapeutic delivery. This Research Topic explores the latest advancements and interdisciplinary efforts driving the clinical transformation of bioactive materials in bone repair applications.

Bone tissue engineering is evolving rapidly, with innovative biomaterial scaffolds and advanced 3D-printing techniques being explored to enhance bone regeneration and provide structural support for treating critical-sized bone defects.

In the article “*Biomaterial Scaffolds Regulate Macrophage Activity to Accelerate Bone Regulation*”, Liu et al. review design strategies for biomaterial scaffolds that optimize macrophage function to enhance bone healing. It emphasizes the role of M2 macrophages in tissue repair and inflammation resolution and discusses the importance of macrophages throughout bone healing stages. Scaffold parameters like biocompatibility, degradation rate, and surface characteristics are crucial for modulating macrophage polarization. Despite promising approaches, inconsistent findings between studies highlight the need for further research. Current clinical applications remain inadequate for severe bone defects, emphasizing the need for precise scaffold designs that support endogenous bone regeneration.

Pei et al. examine how scaffold microstructure influences the controlled release of bioactive factors crucial for bone healing. Key findings include that microstructure, especially pore size and porosity, significantly affects release rates, with 400-micron pores and 70%–80% porosity providing optimal outcomes. Covalently bound factors and nanoparticle encapsulation helped minimize burst-release, offering more stable, sustained delivery. The study underscores the importance of scaffold design, including loading methods and release mechanisms, for effective bone regeneration and suggests future advancements should incorporate secondary mechanical and differentiation properties for improved therapies.

Addressing large bone defects often involves the use of biocompatible scaffolds to enhance both bone regeneration and vascularization. Traditional titanium implants, while mechanically stable, can lack bioactivity essential for bone integration. Wang et al. designed a bioactive scaffold by integrating adipose-derived mesenchymal stem cells (ADSCs) and platelet-rich plasma (PRP) into a 3D-printed porous titanium alloy (PTI) scaffold. This scaffold, termed PTI/AP, leverages PRP’s release of growth factors like PDGF and VEGF, which facilitate angiogenesis and support osteogenic differentiation. *In vitro* tests confirmed that the PTI/AP scaffold promoted both cell proliferation and differentiation, with ADSCs exhibiting enhanced viability within the PRP matrix. Further *in vivo* studies in rabbit femoral defects revealed accelerated bone regeneration and vascularization, as evidenced by increased expression of osteogenic markers and improved micro-CT analysis. The dual-function scaffold offers a promising solution for bone defect treatments, combining structural support with biological enhancement for orthopedic applications.

Bone repair scaffolds for critical-sized defects ideally need both osteoinductive capabilities and imaging properties for monitoring. Silicon and gadolinium co-doped hydroxyapatite/poly(lactic-co-glycolic acid) (Si-Gd-HA/PLGA) scaffolds address these requirements by combining bone-inductive potential with magnetic resonance imaging (MRI) compatibility. Xie et al. synthesized Si and Gd co-doped HA nanoparticles via hydrothermal methods, incorporating these into PLGA to develop multifunctional scaffolds. Optimal doping concentrations of 0.8% silicon and 1.5% gadolinium improved osteogenic gene expression, alkaline phosphatase activity, and calcium deposition *in vitro*. The scaffold’s Gd content also enabled MRI visualization, providing a non-invasive imaging option for real-time monitoring. The 1.5Gd-Si-HA/PLGA scaffold displayed dose-dependent MRI enhancement and osteogenic effectiveness, offering a promising dual-functional material for orthopedic applications, integrating bone regeneration with imaging support for clinical monitoring.

Bone tissue engineering increasingly employs 3D-printed biomaterials to address large bone defects, particularly in femoral and tibial applications. This systematic review and meta-analysis, by Sagar et al., evaluates the efficacy of 3D-printed scaffolds in animal models for repairing critical-sized femoral and tibial defects. The analysis includes 37 studies from 2013 to 2023, assessing composite scaffold properties like porosity (optimal at >50% with 300–400 µM pore size) and their effects on bone volume, trabecular thickness, and structural integrity. Ceramic-based composites, especially those with engineered macro-channels, displayed the highest bone regeneration capacity in femoral and tibial defects. Findings suggest that these 3D-printed scaffolds, particularly those with calcium phosphate or bioactive glass bases, offer promising alternatives to conventional implants by enhancing bone tissue regeneration and structural support in orthopedic applications.

Recent advancements in biomaterials for orthopedic applications are driving the development of innovative infection-resistant and osteogenic solutions, including modified PEEK implants, dual-coated titanium surfaces, and bioactive bone substitutes, to improve bone regeneration and combat infections.

In the article “*Research Progress and Future Prospects of Antimicrobial Modified Polyetheretherketone (PEEK) for the Treatment of Bone Infections*”, Zhang et al. explore advancements in PEEK as a promising alternative to traditional bone cements. PEEK’s high rigidity, malleability for implants, and biological inertness reduce bacterial adhesion and promote osseointegration. Innovations include antibiotic-loaded PEEK, metal ion integration for antimicrobial effects, and 3D-printed textured surfaces to combat bacteria. The goal is to develop sustained-release systems delivering antibiotics directly to infection sites, minimizing systemic side effects and improving infection control. While PEEK shows significant potential, further research is needed to optimize antibacterial efficacy and ensure seamless integration.

The study by Huang et al. explores the enhancement of poly-ether-ether-ketone (PEEK) implants using a self-assembling graphene oxide (GO) coating to boost their antibacterial and osteogenic properties for orthopedic applications. By sulfonating PEEK, researchers developed a porous structure that facilitates GO attachment. While PEEK alone possesses bone-like mechanical properties, it lacks bioactivity and antibacterial features. The GO-coated PEEK composite demonstrated impressive antibacterial performance, reducing bacterial colonies by up to 94%, and significantly improved osteoblast adhesion and proliferation. These findings suggest that the GO-enhanced PEEK implants hold great potential as infection-resistant, osteogenic solutions for orthopedic treatments.

In a retrospective study of 163 patients, Su et al. evaluate the efficacy of a one-stage treatment combining antibiotic-loaded calcium sulfate with autologous bone graft for limb-localized osteomyelitis. The results indicate a significant reduction in infection recurrence rates, minimizing the need for prolonged systemic antibiotic therapy. However, the study identifies increased risk factors for recurrence, including a history of smoking, lower education levels, concurrent flap surgery, and infections related to open injuries. These findings emphasize the importance of careful patient selection and personalized management strategies to optimize outcomes when using this combined approach for osteomyelitis treatment.

In this systematic review, Parizi et al. evaluate the effectiveness of dual-coating titanium implant surfaces with Strontium (Sr) and Silver (Ag) to enhance antibacterial and osteogenic properties. Analyzing 17 *in-vitro* studies, the findings reveal that Sr/Ag dual coatings effectively inhibit *Escherichia coli* and *Staphylococcus* growth. Silver’s antibacterial action is attributed to its disruption of bacterial cell membranes and oxidative stress induction. Strontium enhances bone cell adhesion, proliferation, and differentiation, promoting bone integration while also modulating Silver release to mitigate cytotoxicity, demonstrating a synergistic effect. Despite these promising results, further *in vivo* and clinical research is necessary to confirm the efficacy and elucidate underlying mechanisms.

Jiang et al. evaluates bioactive degradable bone substitutes for the treatment of osteomyelitis, highlighting materials like bioactive glass, calcium sulfate, calcium phosphates, and synthetic polymers. These substitutes show promise for both treating infections and promoting bone regeneration, especially when combined with antimicrobial agents for localized drug delivery at infection sites. Calcium phosphates, for instance, provide an effective scaffold for bone cell attachment and growth, with potential enhancements from osteoinductive and conductive agents. However, challenges such as inconsistent degradation rates and mechanical instability remain. Further research is necessary to optimize these materials and improve clinical outcomes for osteomyelitis patients.

Advancements in surface modification techniques, such as sandblasting and cold physical plasma (CPP) treatments, are offering promising solutions to improve the bioactivity and integration of titanium implants and bone allografts in orthopedic applications.

In the article “*Manufacture of Titanium Alloy Materials with Bioactive Sandblasted Surfaces and Evaluation of Osseointegration Properties*”, Wang et al. investigates enhancing titanium alloy implants for better bone integration through sandblasting surface modification. Using SEM, X-ray photoelectron spectroscopy, and atomic force microscopy, researchers evaluated the morphology and physicochemical properties of treated titanium surfaces. *In vitro* studies with rabbit osteoblasts and *in vivo* rabbit models demonstrated that sandblasted titanium showed no cytotoxicity, higher osteoblast proliferation, increased ALP activity, and upregulated osteogenesis-related gene expression compared to Ti6A14V controls. The results suggest that sandblasting enhances bioactivity and osseointegration, providing a promising approach to improving titanium implant performance.

Surgical treatments involving bone grafts are essential in managing large bone defects, yet challenges in graft integration and risk of contamination persist. Bone allografts, typically processed to prevent immunogenic reactions, often exhibit lower rates of cellular ingrowth. Cold physical plasma (CPP) offers an innovative approach to improve allograft processing by sterilizing the graft surface while preserving its biological properties. In this study, Fischer et al. optimized CPP parameters, examining various gas mixtures and treatment durations, to enhance osteogenic cell viability and activity. Morphological analyses using synchrotron radiation-based microcomputed tomography (SR-μCT) confirmed that CPP treatment did not alter bone structure, making it viable for clinical application. The optimized CPP parameters (3min CPP treatment using He 0.1% N_2_ gas) notably improved bone cell metabolic and alkaline phosphatase activities, vital for osteogenesis, without compromising graft structure. This study positions CPP as a potential method for safer, more effective bone allograft processing, with significant implications for orthopedic surgical outcomes.

